# Differential Signaling by Protease-Activated Receptors: Implications for Therapeutic Targeting

**DOI:** 10.3390/ijms15046169

**Published:** 2014-04-11

**Authors:** Tejminder S. Sidhu, Shauna L. French, Justin R. Hamilton

**Affiliations:** Australian Centre for Blood Diseases & Department of Clinical Haematology, Monash University, Melbourne 3004, Australia; E-Mails: tejminder.sidhu@monash.edu (T.S.S.); shauna.french@monash.edu (S.L.F.)

**Keywords:** protease-activated receptors, G protein-coupled receptors, cell signaling, platelets, thrombosis

## Abstract

Protease-activated receptors (PARs) are a family of four G protein-coupled receptors that exhibit increasingly appreciated differences in signaling and regulation both within and between the receptor class. By nature of their proteolytic self-activation mechanism, PARs have unique processes of receptor activation, “ligand” binding, and desensitization/resensitization. These distinctive aspects have presented both challenges and opportunities in the targeting of PARs for therapeutic benefit—the most notable example of which is inhibition of PAR1 on platelets for the prevention of arterial thrombosis. However, more recent studies have uncovered further distinguishing features of PAR-mediated signaling, revealing mechanisms by which identical proteases elicit distinct effects in the same cell, as well as how distinct proteases produce different cellular consequences via the same receptor. Here we review this differential signaling by PARs, highlight how important distinctions between PAR1 and PAR4 are impacting on the progress of a new class of anti-thrombotic drugs, and discuss how these more recent insights into PAR signaling may present further opportunities for manipulating PAR activation and signaling in the development of novel therapies.

## 1. Introduction

Protease-activated receptors (PARs) are a unique family of G protein-coupled receptors (GPCRs). There are four PARs in mammals, designated PAR1 through PAR4 based on the order of their discovery. PARs are widely expressed throughout the body and are activated by a subset of serine proteases, most notably thrombin (PAR1, PAR3 and PAR4) [1–6], trypsin and coagulation factor Xa (PAR1, PAR2 and PAR4) [7,8], plasmin (PAR1 and PAR4) [9] activated protein C (PAR1) [10,11], tryptase and matriptase (PAR2) [12–14], and cathepsin G (PAR4) [15]. Given the cleavage-based activation mechanism of PARs, this receptor class exhibits a number of unique features that have impacted on the development of potent and specific antagonists. Furthermore, the significant overlap in expression pattern and endogenous protease activators between the PARs has led to the requirement for a detailed understanding of the differences between the mechanisms of receptor activation and of the intracellular signaling elicited by distinct PARs. Despite the significant similarities within this receptor class, a number of important differences exist between PARs that are expressed in the same place and are activated by the same serine proteases. This review outlines some of the important differences in the activation and signaling of PARs, and discusses how these differences have contributed to the successful clinical development of reagents that target specific PARs and how they might be further exploited in the development of additional novel therapies.

## 2. Exploiting Differences in PAR Activation Mechanisms for Therapeutic Benefit

### 2.1. Distinct Mechanisms of Receptor Activation

**Figure 1.**
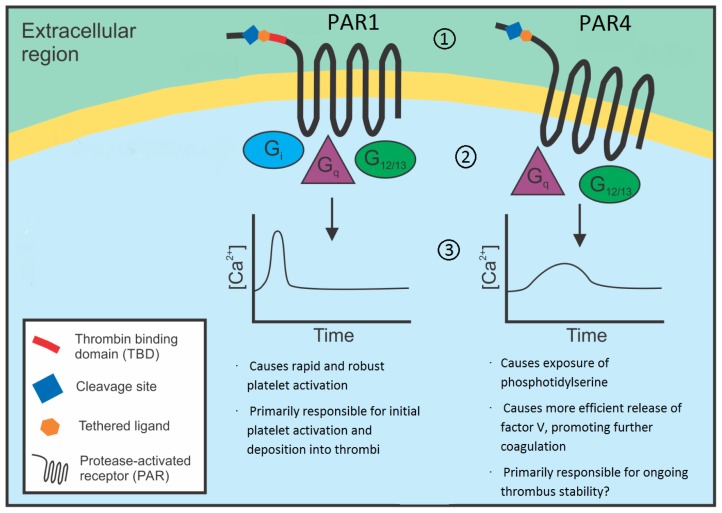
Differential activation of and signaling by PAR1 and PAR4 has implications for drugs targeting thrombin-induced platelet activation. Thrombin activates both PAR1 and PAR4 on human platelets, but with several important distinctions that have important implications for the targeting of PARs as anti-platelet agents. (1) Thrombin activates PAR1 with ~30 fold greater sensitivity than PAR4, in large part due to the presence of a thrombin-binding domain (TBD; red line) in the amino terminal of PAR1 that facilitates thrombin binding and site specific cleavage and receptor activation; (2) Distinct G protein coupling between PAR1 and PAR4 facilitates different intracellular signalling events; (3) Contrasting kinetics of calcium signaling occur following PAR1 and PAR4 activation, with PAR1 activation inducing a rapid rise in intracellular calcium compared with the slower but more prolonged calcium increase induced by PAR4. As a result of these differences, PAR1 antagonists have undergone clinical development as antiplatelet drugs because they target the high affinity thrombin receptor. Whether drugs that block the divergent functions of PAR4 will provide distinct utility as anti-platelet agents remains to be determined.

Each of the four PARs is activated via site-specific serine protease cleavage of the extracellular amino terminal of the receptor, with some minor, albeit important, distinctions in each case. PAR1 is a 425 amino acid seven transmembrane-spanning domain GPCR with a thrombin cleavage site between residues 41 and 42 (LDPR^41^/^42^SFLLRN) [5]. Following receptor cleavage, PAR1 essentially functions as a peptide receptor that carries its own ligand, as the newly created amino-terminus, ^42^SFLLRN^47^, self-activates the receptor by binding to the second extracellular loop in the traditional manner of a peptide GPCR [3]. A hirudin-like thrombin binding domain is present between residues 53 and 64 (DKYEPF) and is the primary binding site between PAR1 and the anion-binding exosite I of thrombin, promoting efficient receptor cleavage [3]. For this reason, PAR1 is a high affinity thrombin receptor and responds to low nanomolar concentrations of the enzyme (Figure 1). In comparison, the other two thrombin-responsive PARs, PAR3 and PAR4, provide significantly weaker responses to thrombin. Indeed, there is scant evidence that PAR3 directly mediates thrombin signaling in cells. Rather, PAR3 appears to function largely as a co-factor to enhance cleavage and activation of PAR4 [16]. In the case of PAR4, the weaker thrombin-mediated response is due in large part to the absence of a thrombin binding domain in the amino terminal of this receptor [2] (Figure 1). In heterologous expression systems or isolated primary cells, PAR1 is approximately 30 times more responsive to thrombin than is PAR4 [16]. As a consequence, cell types expressing multiple thrombin-responsive PARs (e.g., both PAR1 and PAR4 on platelets, endothelial cells, and vascular smooth muscle; all of PAR1, PAR3, and PAR4 on cardiomyocytes and neurons) have a high affinity thrombin receptor (PAR1) a lower affinity thrombin receptors (PAR4) and/or a cofactor (PAR3). This difference in receptor affinity for the activating protease has been exploited in the development of PAR antagonists as anti-thrombotic agents.

### 2.2. Differences in Receptor Activation Mechanisms Promote the Targeting of PAR1 for the Prevention of Arterial Thrombosis

Arterial thrombosis is the leading cause of mortality and morbidity in the developed world and is vitally dependent on the incorporation of activated platelets into thrombi [17,18]. As a result, there has been much research into understanding platelet activation mechanisms for the development of improved anti-platelet therapies. Current thinking suggests platelet activation during arterial thrombosis occurs in three stages: adhesion, activation and consolidation [19]. These stages have distinct functions in the formation and stabilisation of the thrombus, although drugs that impair the activation stage of platelet-thrombus formation have been by far the most successful anti-platelet agents to date. Adhesion of platelets at sites of damaged blood vessels is mediated by a series of surface glycoprotein receptors present in high quantities on the surface of platelets, most notably the GPIb/V/IX and GPIIb/IIIa complexes [19]. Once these adhesion receptors localize platelets to the site of vascular injury, platelet activation occurs and is largely mediated by three substances: ADP, thromboxane A2 (TxA2) and thrombin [19]. These three agents co-ordinately activate localized platelets resulting in the formation of a platelet-rich thrombus and, of these, thrombin is easily the most potent activator and the only one not targeted by current anti-platelet drugs. Specifically, current long-term anti-platelet therapies for the prevention of arterial thrombosis involve either blockade of TxA2 production (*i.e.*, aspirin) or inhibition of ADP-mediated platelet activation (e.g., clopidogrel). Given the potent effects of thrombin on platelet activation and the well-known anti-thrombotic effects of thrombin inhibitors, drugs that prevent thrombin-induced platelet activation are a promising target for novel anti-thrombotics.

Human platelets express both PAR1 and PAR4 [1,2,20]. However, as discussed above, PAR1 exhibits significantly higher affinity for thrombin due to the presence of a thrombin binding domain (Figure 1). Exploiting the different affinities of these two co-expressed thrombin receptors has resulted in a strong focus on the development of PAR1 antagonists, and two such agents, atopaxar and vorapaxar, entered Phase 3 trials for the prevention of arterial thrombosis [21]. Both drugs are reversible, competitive PAR1 antagonists. Atopaxar demonstrated safety in a Phase 2 trial [22]. Vorapaxar recently completed Phase 3 trials [23], but provided an insignificant net clinical benefit largely due to an increase in intracranial bleeding observed in participants with a history of stroke [21,23–25]. These recent clinical findings indicate more needs to be done to understand the role of platelet PARs in arterial thrombosis. Whereas much of the research on platelet PARs has focused on PAR1, PAR4 has largely been ignored. While it is known both PAR1 and PAR4 are capable of fully activating platelets [2,20] it is less clear why both are present on the cell. It has been theorised that PAR4 acts as a “back up” in case of PAR1 failure. Another, arguably more attractive, hypothesis is that the two receptors have divergent functions in platelet activation. The distinct activation mechanisms and intracellular signaling events elicited by these two thrombin receptors, as discussed above, supports this latter hypothesis and suggests that targeting PAR1 and PAR4 may provide disparate clinical effects (see Section 3.2, below).

### 2.3. Differences in Receptor Activation Mechanisms Promote the Targeting of PAR2 for the Prevention of Histamine-Independent Itch

PAR2 is the most functionally distinct receptor in the PAR family: it is the only PAR insensitive to thrombin and is activated by a number of serine proteases not thought to productively cleave any other PAR (e.g., tryptase, matriptase). Consequently, PAR2 has largely been studied in distinct cell types and disease processes than the other PAR family members, and may provide a therapeutic target in a range of different settings. For example, the expression of PAR2 on inflammatory cells and the robust activation of PAR2 by mast cell tryptase [12,26], has generated significant interest in targeting PAR2 in immune-based pathologies, and inhibition of PAR2 is currently under consideration for the prevention of histamine-independent itch [27–29].

## 3. Exploiting Differences in PAR-Mediated Signaling for Therapeutic Benefit

### 3.1. Distinct Intracellular Signaling Events

Activation of PARs induces a variety of cell signaling. Of the four major Gα protein subclasses, all of Gq, Gi and G12/13 have been demonstrated to signal following activation of any of PAR1, PAR2, and PAR4 [30–32], although the coupling of PAR1 and PAR4 to Gi remains controversial [31,33–35] and whether or not PAR3 directly signals via G proteins is unclear: human PAR3 has been shown to signal autonomously via Gq [36] but mouse PAR3 fails to transduce an intracellular signal [1,37,38]. For PAR1, early studies showed receptor activation inhibits cAMP accumulation via Gi, and stimulates phospholipase C (PLCβ)-mediated hydrolysis of phosphotidylinosotol 4,5-bisphosphate (PIP_2_) and generation of inositol 1,4,5-trisphosphate (IP_3_) and diacylglycerol (DAG) [31]. Ca^2+^ mobilization is induced via Gq [31], and is important for the function of several key signaling enzymes, including protein kinase C (PKC), phospholipase A_2_, and calpain [4,39]. In contrast to these divergent Gi and Gq signaling pathways, concomitant Gq and Gi signaling in response to PAR1 activation appears to stimulate ERK1 and ERK2 [40,41]. Direct coupling of PAR1 to G12/13 leads to activation of Rho GEFs, induction of cytoskeletal changes, and PLC activation [30,42–45]. There is no analogous direct evidence linking PAR2 to G proteins, although PAR2 activation increases second messenger responses suggestive of Gq, Gi, and G12/13 signaling [45]. Finally, PAR4 appears to couple to Gq and G12/13 [32,45], and possibly Gi [33], in a similar repertoire to that observed for PAR1.

### 3.2. Will Differences in Intracellular Signaling Events Promote the Targeting of PAR4 for the Prevention of Arterial Thrombosis?

The coupling of PARs to distinct G protein effectors represents a clear point of difference within this receptor class and provides opportunities for disparate effects of therapeutic targets. Of particular relevance to the first therapeutic strategy targeting PARs is the temporal difference in intracellular calcium signaling elicited by thrombin activating PAR1 *versus* PAR4. Specifically, PAR1 activation leads to a rapid but transient rise in intracellular calcium in a range of cell types, whereas PAR4 activation induces a calcium signal which is slower in onset but is markedly more sustained [46] (Figure 1). This distinction between intracellular signaling events may be relevant to the use of PAR antagonists as anti-platelet drugs. One key platelet activation event reliant on sustained intracellular calcium signaling is the exposure of phosphotidylserine (PS) on the outer membrane surface of the platelet—the hallmark feature of the platelet procoagulant response in which coagulation factors assemble on the modified platelet surface and thrombin generation occurs [47]. This may be of significance in the setting of arterial thrombosis, because the platelet procoagulant response is critical for coagulation-dependent fibrin formation. Since arterial thrombi are essentially composed of activated platelets and fibrin, inhibition of platelet activation in the absence of inhibition of platelet procoagulant activity may allow distinction between the prevention of platelet deposition (more important for thrombosis) and the prevention of fibrin formation (more important for haemostasis). To this end, selective PAR inhibition may have distinct utility in arterial thrombosis compared to direct thrombin inhibitors such as hirudin, which completely inhibit thrombin and consequently fibrin [48]. However, despite this prediction, it remains unclear which of PAR1 or PAR4 are the main drivers of platelet procoagulant activity, with evidence for both PAR1 [47] and PAR4 [49] as the main driver of thrombin-stimulated platelet procoagulant activity. The paucity of PAR4 antagonists has not helped in this regard, while the use of genetically-modified mouse models is of limited assistance: although a previous study showed that arterial thrombi in PAR4−/− mice have a 10 fold decrease in platelets without any difference in fibrin levels when compared with wild-type controls [50], the significant difference between human and mouse platelet PARs (mouse platelets do not express PAR1) suggests these findings are not directly translatable to humans. As a result, robust and specific PAR4 antagonists, such as those currently being pursued [51], are required to further examine the possibility that PAR1 and PAR4 on human platelets perform separate functions, and to determine whether selective inhibition of these two thrombin receptors will provide distinct utility in the prevention of arterial thrombosis.

Whereas the type and extent of effector signals generated in response to PAR activation provides one level of signaling divergence exploitable for therapeutic gain, more recent developments in platelet PAR signaling indicate more complicated mechanisms also exist. One very recent observation reports that miRNA-based regulation of platelet protein expression varies according to race, and that this variation is significant enough to impact on overall platelet function [52]. Specifically, Edelstein and colleagues [52] showed that platelets from black patients responded significantly more sensitively to PAR4 activation, while responses to all other platelet-activating agents examined were not different. In an elegant series of experiments, the authors showed that miRNA regulation of the expression of phosphotidylcholine transfer protein (PC-TP) underlies this functional difference. These findings define an additional layer of complexity with respect to PAR-mediated signaling and highlight yet another important difference within the receptor class. Perhaps most importantly, these findings open up additional possibilities for exploiting differences in PAR-mediated signaling and provide critical considerations for the use of existing and any novel PAR-targeted therapeutics.

### 3.3. Will Differences in Effector Mechanisms Promote the Selective Targeting of PAR1-Mediated Signaling for the Prevention of Vascular Inflammation?

One exciting recent development in the field of PAR signaling is the discovery of biased agonism of PAR1 [53–55]. Although signaling bias is a well-known phenomenon for many GPCRs, a mechanism by which different proteases could induce distinct intracellular signals despite promoting receptor activation by revealing the same internal peptide sequence remained elusive. However, recent work from several laboratories has revealed how such biased agonism of PARs might occur [53,54,56–62]. Perhaps the most significant and clinically translatable findings in this regard to date relate to the differential effects of thrombin and activated protein C (APC) activation of PAR1 on vascular endothelial cells. It has long been observed that thrombin mediates a well-defined set of pro-inflammatory events in many cell types, including an increase in vascular endothelial permeability, via PAR1 [63,64]. In striking contrast, APC appears to exert precisely the opposite effects, being in general anti-inflammatory and preventing endothelial permeability in response to acute inflammatory challenges, but also via PAR1 [10,65,66]. The confounding ability of PAR1 to mediate both pro- and anti-inflammatory effects has been a topic of significant debate in recent years. In part, this is because although both thrombin and APC are capable of cleaving and activating PAR1, thrombin induces PAR1 cleavage and subsequent intracellular signaling in vascular endothelial cells with ~10,000-fold greater efficiency than does APC [67]. In addition to a lack of understanding how different proteases activating the same receptor could induce opposing cellular effects, this finding questioned exactly when and how cells would experience a 10,000-fold greater concentration of one enzyme *versus* another. One potential explanation for this discrepancy came in the form of receptor partitioning [53]. Specifically, Trejo and colleagues showed that compartmentalization of PAR1 into caveolar microdomains was required for APC- but not thrombin-induced signaling and subsequent protection of endothelial barrier function. Furthermore, they showed that thrombin and APC promote distinct intracellular signaling events downstream of PAR1 (thrombin causing the typical Gi-mediated signaling; APC causing β-arrestin recruitment and activation of the dishevelled-2), as well as a distinct pattern of receptor internalization (thrombin, but not APC, causing receptor phosphorylation, internalization, and sorting to lysosomes). Whether or not such remarkable protease-dependent selectivity exists for the other PARs remains unknown, but is clearly of significant interest. Regardless, however, these intriguing findings lend themselves to the possibility of selective targeting of PAR1-mediated effects. Given the pro- and anti-inflammatory effects mediated by PAR1 activation by thrombin and APC respectively, one attractive possibility is the selective inhibition of thrombin-mediated PAR1-dependent vascular endothelial inflammatory effects in conditions such as sepsis. APC remains the sole treatment option for septic patients. Despite this, the effects of a global PAR1 deficiency in animal models of sepsis remain controversial [68–70], perhaps in part due to the combined effects of both thrombin and APC in this model. Selective inhibition of thrombin-mediated PAR1 activation may prevent the clear pro-inflammatory effects of thrombin while allowing the cytoprotective effects of APC treatment in this condition. Such a case further highlights how a detailed understanding of the distinct activation mechanisms and cell signaling events downstream of PARs may yield opportunities for novel drug therapies.

## 4. Exploiting Differences in PAR-PAR Interactions for Therapeutic Benefit

### 4.1. Distinct Receptor Dimerization Events

A number of studies have shown that PARs form both homodimers and heterodimers that in some instances act to significantly regulate receptor function and signaling, thereby providing an additional possibility for targeted therapeutic intervention (recently reviewed in detail [71,72]). The transactivation of PARs is a well-known phenomenon [73,74], as is the cofactoring function of PARs, most notably that of PAR3 enhancing the cleavage and activation of PAR4 [16]. However, the more recent identification and characterization of PAR dimers has the potential to impact both on current therapeutic strategies that target PAR monomers, as well as on future therapeutic strategies that may aim to specifically target PAR dimers in certain pathologic conditions.

There is evidence that each of the four PARs form heterodimers, with the best-defined being PAR1-PAR4 [75] and PAR1-PAR2 [69] and that may have important implications for the treatment of arterial thrombosis and sepsis, respectively. Given their potential impact on current and future therapeutic approaches, we focus on these particular PAR heterodimers below.

### 4.2. Targeting PAR1-PAR4 Heterodimers for the Prevention of Arterial Thrombosis

PAR1 and PAR4 form stable heterodimers and co-immunoprecipitate in protein lysates from human platelets and mouse fibroblasts [75]. A pharmacological strategy that prevents association of PAR1 and PAR4 impairs signaling via either receptor [75], suggesting reciprocal assistance in the cleavage and activation of these two thrombin receptors. Given that pharmacological disruption of the PAR1-PAR4 interaction effectively prevented carotid artery occlusion in an *in vivo* thrombosis model, this finding has potential implications regarding the inhibition of platelet PARs for the prevention of arterial thrombosis. This work demonstrates one potential advantage of inhibiting PAR1-PAR4 heterodimerization *versus* the more standard receptor antagonist approaches utilized by current PAR antagonists that act to prevent receptor-agonist association. Such an approach has been achieved in proof-of-concept studies using pepducins—lipid moieties attached to peptides that mimic the cytoplasmic loops of GPCRs and thereby modulate signal transference from GPCRs to G proteins [76]. Pepducin-based inhibition of PAR signaling has been shown to effectively modulate GPCR activity using *in vivo* animal models of thrombosis and may provide the advantage of more effectively inhibiting thrombin-induced platelet activation (blocking both PAR1 and PAR4) than is currently afforded (PAR1 inhibition only), although the specificity of such an approach remains unknown.

PAR4 has more recently been shown to form homodimers that also appear vital for normal signaling. Using bimolecular fluorescence complementation (BiFC) and bioluminescence resonance energy transfer (BRET), PAR4 homodimers were mapped to transmembrane helix 4 [77]. While mutations in single residues in this area of the receptor were insufficient to disrupt dimerization, mutations across a series of hydrophobic residues in transmembrane domain 4 reduced PAR4-mediated calcium mobilization in response to a PAR4-activating peptide [77]. This recent work suggests disrupting PAR4 homodimer formation and/or function provides a further approach for novel anti-platelet agents that target thrombin-induced platelet activation.

### 4.3. Targeting PAR1-PAR2 Heterodimers for the Treatment of Sepsis

As discussed above, current therapies for the treatment of sepsis are limited and improved approaches have been keenly sought for decades. Given that PARs are strongly activated by coagulation proteases, they have long been thought to act at the intersection of coagulation and inflammation. As a consequence, there was much early interest in targeting PARs in the setting of sepsis. Despite this, the evidence from animal models that targeting PAR1 and/or PAR2 might be useful in septicemia is inconclusive [68–70]. Based on more recent studies, one possibility is for a more targeted approach of inhibiting PAR1-PAR2 heterodimer formation/function for the treatment of sepsis.

PAR1 and PAR2 co-immunoprecipitate and single-cell confocal fluorescence resonance energy transfer (FRET) imaging confirms the close association of these receptors [69]. Stable PAR1-PAR2 dimers are present during the progression of sepsis [69]. Furthermore, PARs appear to switch from performing a largely proinflammatory response to a more protective response during sepsis. Although the role of PAR1-PAR2 heterodimers have not yet been fully elucidated in this setting, the formation of PAR1-PAR2 complexes are endotoxin-dependent and appear to promote cytoprotective effects in endothelial cells [69]. These findings suggest that therapeutics designed to selectively manipulate PAR1-PAR2 heterodimers may be beneficial in attenuating sepsis progression.

### 4.4. Targeting PAR3 Heterodimers for Therapeutic Benefit

The discovery of PAR3 heterodimers may have uncovered previously unknown roles of this understudied receptor. For example, PAR3 has been shown to form heterodimeric complexes with PAR1 [78,79] and PAR2 [79] that may function to promote cytoprotective signaling mechanisms. Specifically, APC appears to inhibit podocyte apoptosis via proteolyic activation of PAR3 and subsequent dimerization with PAR2 (in humans) or PAR1 (in mice) [78]. This protective effect of PAR-mediated APC signaling occurred independently of EPCR but was dependent upon PAR3 heterodimer formation, and resulted in marked protection against podocyte injury and proteinuria [78]. Aside from revealing novel functions for the enigmatic PAR3, these recent advances may provide rationale for the design of cell-targeted cytoprotective therapies.

## 5. Conclusions

PARs are a unique set of four GPCRs that, as a receptor class, display distinctive modes of receptor activation, signal transduction, and receptor desensitization/resensitization. In addition, within this class of receptor, further divergent mechanisms of these processes exist. These distinctions have been exploited in the development of upcoming therapeutics, with the specific inhibition of the high affinity thrombin receptor on human platelets as an anti-thrombotic approach providing a strong example of how a detailed understanding of the mechanisms of receptor activation and signal transduction are important for appropriate clinical endpoints. In addition, more recent discoveries have highlighted: (1) how the same PAR responds differently to distinct activators to promote opposing effects within the same cell type; (2) how miRNA-based regulation of protein expression varies according to race and impacts on PAR function, and; (3) how the occurrence of various PAR homo- and heterodimers controls overall PAR-mediated effects in a number of distinct pathological conditions. These remarkable recent findings are likely to impact current strategies designed to target PAR signaling. More intriguingly, they may provide additional opportunities for the development of specific drug therapies targeting a range of pathologies in which PARs and their cognate proteases are involved.
